# Solution structure and dynamics of anti-CRISPR AcrIIA4, the Cas9 inhibitor

**DOI:** 10.1038/s41598-018-22177-0

**Published:** 2018-03-01

**Authors:** Iktae Kim, Migyeong Jeong, Donghyun Ka, Mookyoung Han, Nak-Kyoon Kim, Euiyoung Bae, Jeong-Yong Suh

**Affiliations:** 1 Department of Agricultural Biotechnology and Research Institute of Agriculture and Life Sciences, Seoul National University, Seoul, 08826 South Korea; 2 Advanced Analysis Center, Korea Institute of Science and Technology, Seoul, 02792 Korea; 3 Institute for Biomedical Sciences, Shinshu University, Minamiminowa, Nagano 399-4598 Japan

## Abstract

The bacterial CRISPR-Cas system provides adaptive immunity against invading phages. Cas9, an RNA-guided endonuclease, specifically cleaves target DNA substrates and constitutes a well-established platform for genome editing. Recently, anti-CRISPR (Acr) proteins that inhibit Cas9 have been discovered, promising a useful off-switch for Cas9 to avoid undesirable off-target effects. Here, we report the solution structure and dynamics of *Listeria monocytogenes* AcrIIA4 that inhibits *Streptococcus pyogenes* Cas9 (*Spy*Cas9). AcrIIA4 forms a compact monomeric αβββαα fold comprising three antiparallel β strands flanked by three α-helices and a short 3_10_-helix. AcrIIA4 exhibits distinct backbone dynamics in fast and slow timescales at loop regions that form interaction surfaces for *Spy*Cas9. In particular, the β1–β2 loop that binds to the RuvC domain of *Spy*Cas9 is highly mobile, and the β1–β2 and α2–α3 loops that bind to the RuvC and C-terminal domains of *Spy*Cas9, respectively, undergoes conformational exchanges in microsecond-to-millisecond time scales. AcrIIA4 binds to apo-*Spy*Cas9 with *K*_D_ ~4.8 μM, which compares to *K*_D_ ~0.6 nM for AcrIIA4 binding to sgRNA-bound *Spy*Cas9. Since the binary complex between AcrIIA4 and *Spy*Cas9 does not compete with the target DNA binding, it can effectively disable the Cas9 nuclease activity by forming a tight ternary complex in the presence of sgRNA.

## Introduction

The clustered regularly interspaced short palindromic repeats (CRISPR) and CRISPR-associated (Cas) proteins constitute a defense system in bacteria and archaea, providing a form of immunological memory against foreign genetic elements^1,2^. Upon infection, the bacterial host cell activates the CRISPR-Cas system to integrate short segments of the invading nucleic acids into the CRISPR loci. The integrated DNA sequences, also known as spacers, are transcribed into CRISPR RNAs that guide a single protein effector or a multi-protein effector complex to degrade the complementary sequences in foreign genes upon subsequent invasions. The CRISPR-Cas system is categorized into two classes, six types and thirty-three subtypes according to different organizations of signature *cas* genes^3^. *Streptococcus pyogenes* Cas9 (*Spy*Cas9) belongs to the subtype II-A of the Class 2 CRISPR-Cas system. *Spy*Cas9 has been extensively investigated and widely applied to genome editing due to its simplicity in generating double-strand DNA breaks at desired sites targeted by single-guide RNA (sgRNA)^4–6^.

Bacteria and phages have long been engaged in an arms race, developing a variety of arsenal to compete for invasion and defense^7^. Recently, anti-CRISPR (Acr) proteins that neutralize Cas9 activity have been discovered in phages targeting pathogenic bacterial strains^8,9^. Among them, AcrIIA2 and AcrIIA4 encoded by *Listeria monocytogenes* prophages have shown cross-strain inhibitory effects against *Spy*Cas9, highlighting their potential as an off-switch for Cas9 for temporal and spatial control of genome editing. Recent structures of the AcrIIA4‒*Spy*Cas9‒sgRNA complex obtained by X-ray crystallography and electron microscopy show that *Spy*Cas9 binds to AcrIIA4 via the protospacer adjacent motif (PAM) interaction site and the RuvC domain^10–12^. Here we report the first solution structure and dynamics of AcrIIA4 using NMR spectroscopy. Backbone dynamics from ^15^N NMR relaxation data demonstrates that the binding interfaces for *Spy*Cas9 exhibit fast internal motions and slow conformational exchanges in variable timescales. Further, we show that AcrIIA4 binds to *Spy*Cas9 in the absence of sgRNA with an equilibrium dissociation constant (*K*_D_) of ~4.8 μM, which can associate with guide RNA to form a tight ternary complex, leading to the loss of Cas9 nuclease activity.

## Results and Discussion

### Structural description of AcrIIA4 determined by NMR spectroscopy

**Figure 1:**
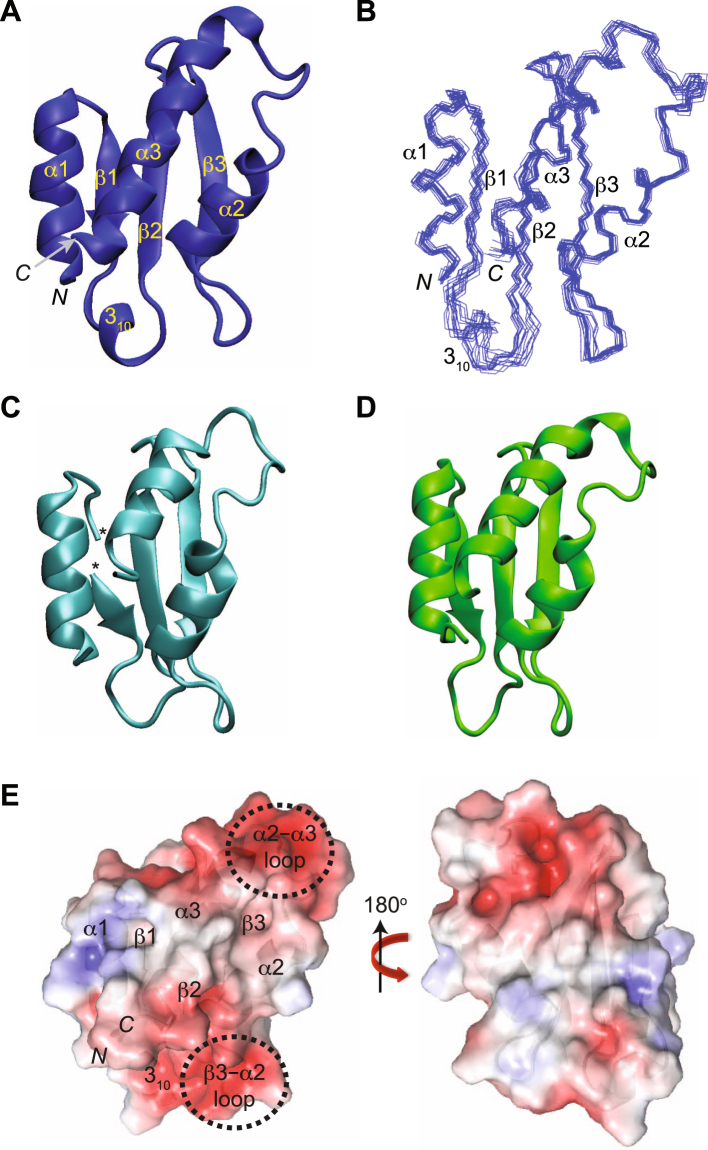
Structures of AcrIIA4 as ribbon and surface representations. (**A**) Solution structure of AcrIIA4 in the free state. Secondary structures, as well as N- and C-termini are annotated. (**B**) Superposition of the backbone atoms of the final 20 simulated annealing structures of AcrIIA4. The structures are best-fit superposed against well-ordered secondary structures between residues 2–13, 16–19, 29–33, 40–44, 50–59, and 68–85. (**C**) AcrIIA4 from the crystal structure of the AcrIIA4–*Spy*Cas9–sgRNA complex (PDB code 5XBL). (**D**) AcrIIA4 from the crystal structure of the AcrIIA4–*Spy*Cas9–sgRNA complex (PDB code 5VW1). (**E**) Front and backside view of molecular surface representations of AcrIIA4 color-coded by electrostatic potential, ±5 kT. AcrIIA4 on the left panel is drawn in the same orientation as in (**A**).

AcrIIA4 (a.a. 1‒87) exists as a monomer in solution, and exhibits a well resolved ^1^H–^15^N HSQC NMR spectrum that is typically observed in compact folded proteins (Figures S1 and S2). We have determined the solution structure of AcrIIA4 using heteronuclear triple-resonance NMR spectroscopy. Backbone and side chain assignments were obtained using a suite of three-dimensional heteronuclear correlation NMR spectroscopy experiments. The backbone chemical shifts were completely assigned except for Thr28 that did not show backbone amide resonance in the 2D ^1^H–^15^N HSQC spectrum. Ser20, Ser24, Gln29, Glu47, and Gln65 did not show backbone amide resonances, but their aliphatic and carbonyl groups could be assigned from their sequential connectivities. The ^1^H assignment data covered 602 out of 646 ^1^H atoms excluding those in exchangeable hydroxyl and amino groups, which amounts to the 93.2% completeness of ^1^H assignment. Three-dimensional ^13^C-separated NOE and ^15^N-separated NOE restraints were employed for the structure calculation using the Xplor-NIH program^13^. Residual dipolar couplings (RDCs) were measured in 10 mg/ml of *pf1* phage alignment medium. The structure was determined using 1,782 NMR restraints including 1,504 experimental NOE-based distance restraints, 161 dihedral angle restraints, 79 backbone ^1^D_NH_ RDC restraints, and 38 hydrogen bonding restraints (Table 1). The hydrogen bond restraints were introduced at the final stage of the structure calculation, based on the secondary structural information from NOE and chemical shift data. The hydrogen bond restraints were in perfect agreement with the short-range and medium range NOEs, and dihedral angle restraints. AcrIIA4 is comprised of three antiparallel β strands (β1, 16–19; β2, 29–33; β3, 40–44) and three flanking α helices (α1, 2–13; α2, 50–59; α3, 68–85) in an α1–β1–β2–β3–α2–α3 order (Fig. 1A). In addition, a short 3_10_ helix (22–25) is formed in the loop connecting β1 and β2 strands. Superposition of the backbone atoms for the ensemble of the 20 lowest energy structures of AcrIIA4 demonstrates that overall secondary structures are well defined such that three helices are packed together upon a three-stranded β sheet (Fig. 1B). The connecting loop regions are less well-defined, and the β1‒β2 loop among others shows the largest backbone root-mean-square deviations (RMSD ~1.3 Å).

**Table 1:** Restraints and structural statistics for AcrIIA4.

Experimental restraints	<SA>*
Nonredundant NOEs	1504
Dihedral angles, ϕ/ψ/χ	71/71/19
Hydrogen bonds	38
Residual dipolar coupling, ^1^D_NH_	79
Total number of restraints	1782 (20.5 per residue)
rms deviation from experimental restraints	
Distances (Å) (1504)	0.029 ± 0.001
Torsion angles (°) (161)	0.90 ± 0.10
Residual dipolar coupling *R*-factor (%)^†^	
^1^D_NH_ (%) (79)	1.8 ± 0.2
rms deviation from idealized covalent geometry	
Bonds (Å)	0.002 ± 0
Angles (°)	0.47 ± 0.01
Impropers (°)	0.42 ± 0.01
Coordinate precision (Å)*^‡^	
Backbone	0.61 ± 0.11
Heavy atoms	1.38 ± 0.09
Ramachandran statistics (%)^‡§^	
Most favorable regions	91.2 ± 1.1
Allowed regions	8.8 ± 1.1

^*^For the ensemble of the final 20 simulated annealing structures.

^†^The magnitudes of the axial and rhombic components of the alignment tensor were 7.3 Hz and 0.35, respectively.

^‡^Regions with secondary structures (residues 2–13, 16–19, 29–33, 40–44, 50–59, and 68–85).

^§^Calculated using the program PROCHECK^39^.

The overall backbone fold of free AcrIIA4 in solution was similar to that of the crystal structures determined for the AcrIIA4‒*Spy*Cas9‒sgRNA complexes, indicating that AcrIIA4 does not change the backbone conformation upon binding to *Spy*Cas9‒sgRNA (Fig. 1C and D). The β1 strand was well-defined and the C-terminal α3 helix extended between Glu68 and Glu85 in the solution structure, which was consistent with the crystal structure with higher resolution (Fig. 1D). On the other hand, the β1‒β2 loop formed a 3_10_ helix between Thr22 and Asn25 in the solution structure, but did not exhibit a regular secondary structure in complex with *Spy*Cas9‒sgRNA.

AcrIIA4 features a cluster of aromatic residues that form a compact hydrophobic core via stacking and edge-to-face packing between the β3 strand, and α2 and α3 helices. The tightly packed aromatic side chains result in large ring current effects and unusual chemical shifts, such as Hβ of Glu71 (−0.1 ppm), Hα of Gln65 (0.4 ppm), and Hβ and Hγ of Met77 (0.17 ppm and −0.05 ppm). AcrIIA4 is an acidic protein (pI ~4.2), and the surface electrostatic potential indicates that negative charges are densely populated in the β3–α2 loop, the α2–α3 loop and the beginning of the α3 helix (Fig. 1E). It has been suggested that the negatively charged patches mimic the phosphate groups of nucleic acids that associate with the PAM interaction site of *Spy*Cas9^10–12^, which was also found in AcrF2 proteins inhibiting the subtype I-F effector complex^14^.

### Backbone dynamics reveals characteristic motions at the binding interface

**Figure 2:**
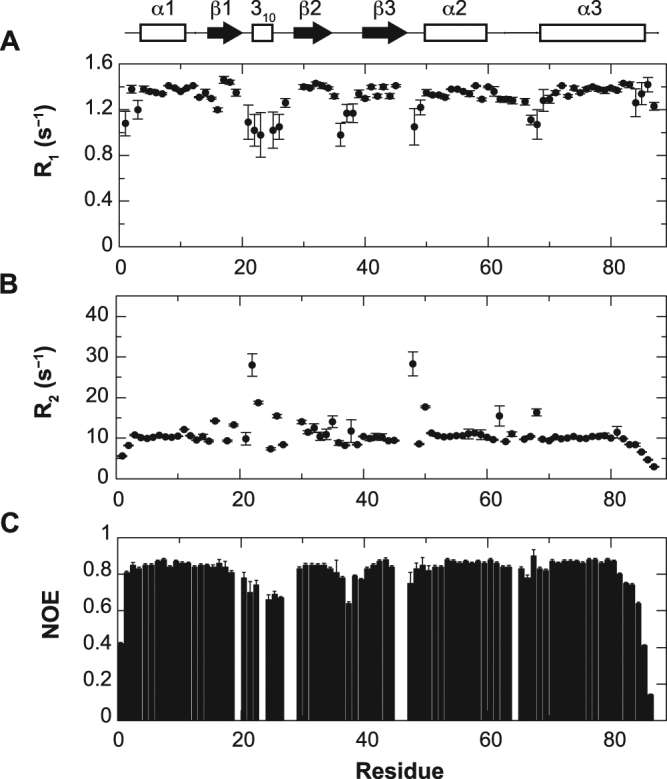
Plots of relaxation parameters of backbone amide groups of AcrIIA4. (**A**) ^15^N R_1_ relaxation, (**B**) ^15^N R_2_ relaxation, and (**C**) ^1^H–^15^N heteronuclear NOE data as a function of residue number. The secondary structures of AcrIIA4 are shown on top as a schematic representation. The relaxation parameters were obtained on an 800 MHz Bruker NMR spectrometer.

^15^N R_1_ and R_2_ relaxation rates and ^1^H–^15^N heteronuclear NOE were measured to characterize the backbone dynamics of AcrIIA4. The rotational correlation time (τ_c_) was calculated as 6.2 ns from the ratio of ^15^N R_2_ and R_1_ relaxation rates in the ordered region, which is typical for the size of AcrIIA4 (~10.2 kDa). Secondary structural regions exhibited large ^1^H–^15^N heteronuclear NOE (>0.8), and uniform ^15^N R_2_ and R_1_ relaxation parameters indicating that the secondary structures were well structured and rigid without internal motions (Fig. 2). On the other hand, loop regions connecting the secondary structures manifested variable dynamic behavior in a wide range of timescales (Fig. 2). Reduced ^1^H–^15^N heteronuclear NOE (<0.8) and ^15^N R_1_ relaxation rates indicated that fast backbone dynamics in ps‒ns timescales was prevalent in the β1–β2 loop (Gly21–Ile27) region. In addition, increased ^15^N R_2_ relaxation rates indicated the presence of slower conformational exchanges in μs‒ms timescales in the β1–β2 loop (Thr22, Asn23, and Ser26), the β3–α2 loop (Asn48 and Ser50) and the α2–α3 loop (Gly62 and Glu68) regions. The conformational exchanges also resulted in complete line broadening of backbone amide resonances in the β1–β2 loop (Ser20, Ser24, Thr28, and Gln29), the β3–α2 loop (Glu47), and the α2–α3 loop (Gln65) regions.

**Figure 3:**
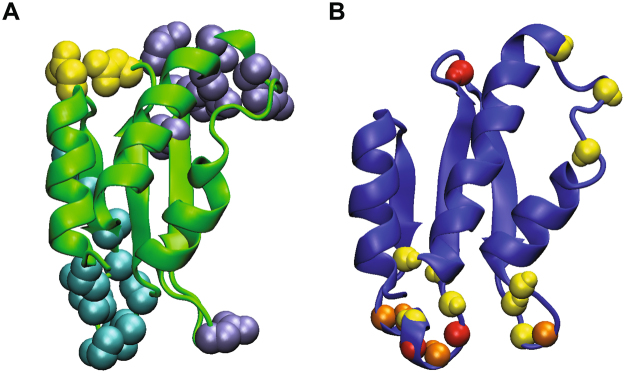
Binding interfaces of AcrIIA4 for *Spy*Cas9‒sgRNA and mobile regions with backbone dynamics. (**A**) Binding interfaces of AcrIIA4 from the crystal structure in complex with *Spy*Cas9‒sgRNA (PDB code 5VW1). AcrIIA4 is shown as a ribbon diagram, and interfacial residues in the complex are shown as a space-filling model. Interfaces for the TOPO domain, the RuvC domain, and the C-terminal domain of *Spy*Cas9 are colored in *yellow*, *cyan*, and *ice-blue*, respectively. (**B**) Mobile regions with backbone dynamics of AcrIIA4 from the solution structure. AcrIIA4 is shown as a ribbon diagram, and backbone amide groups are shown as a space-filling model. Residues with fast internal motions (ps‒ns time scale) and slower conformational dynamics (μs‒ms time scale) are colored in *red* and *yellow*, respectively. Residues with both fast and slower dynamics are colored in *orange*.

It is remarkable that the dynamic loop regions constitute the binding interfaces between AcrIIA4 and *Spy*Cas9 in the crystal structure^10–12^. AcrIIA4 mainly interacts with the TOPO domain, the RuvC domain, and the C-terminal domain (CTD) of *Spy*Cas9. Specifically, AcrIIA4 employs the α1–β1 and β2–β3 loops to interact with the TOPO domain, the β1 strand and the β1–β2 loop to interact with the RuvC domain, and the β2–β3, β3–α2, and α2−α3 loops to interact with the CTD (Fig. 3A). The interfacial loops exhibited significant backbone dynamics in a wide range of time scales. The β1–β2 loop that binds to the RuvC domain of *Spy*Cas9 was highly mobile with fast internal motions in ps‒ns timescales as well as slower conformational exchanges in μs‒ms timescales (Fig. 3B). On the other hand, the β3–α2 loop and the α2–α3 loop that bind to the CTD of *Spy*Cas9 exhibited extensive conformation exchanges in μs‒ms timescales. The binding interface for the TOPO domain appeared less dynamic compared to the other interfaces.

It has been reported that the fast and slow motions characterized by NMR relaxation play an important role in the target search and recognition process of biomolecular interactions^15–18^. It is notable that the dynamic β1–β2 loop of AcrIIA4 directly interacts with the active site of the *Spy*Cas9 nuclease, preventing the substrate DNA binding. *Spy*Cas9 cleaves double-stranded DNA substrates using two nuclease domains, the HNH nuclease domain that processes the target DNA strand complementary to the guide RNA, and the RuvC nuclease domain that processes the non-target DNA strand. The β1–β2 loop of AcrIIA4 extends to the active site of RuvC, and participates in a hydrogen bonding network with catalytic residues, blocking the access of the non-target DNA strand. Meanwhile, the α2–α3 loop that recognizes the PAM interaction site for the target DNA strand manifests slower conformational exchanges. The α2–α3 loop does not directly block the active site of the nuclease, but occludes the binding site of the PAM sequence that is located immediately after the cleavage site of the target DNA strand. The loop dynamics of AcrIIA4 are thus linked to the target recognition and binding, with different time scales between the interaction surfaces.

### AcrIIA4 binds to *Spy*Cas9 in the absence of sgRNA

**Figure 4:**
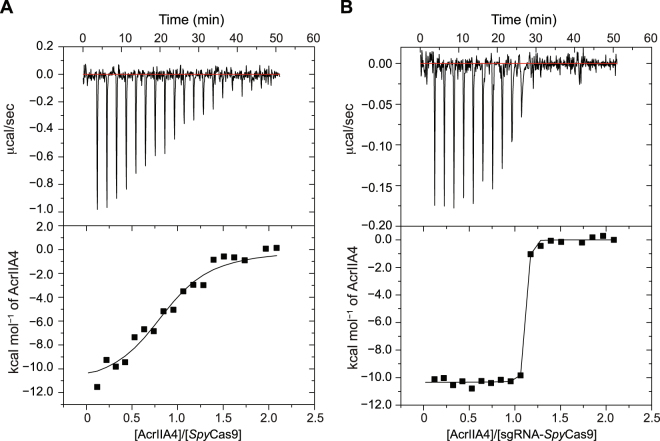
ITC for the interaction (**A**) between AcrIIA4 and *Spy*Cas9, and (**B)** between AcrIIA4 and sgRNA-bound *Spy*Cas9. Raw ITC data (Top panel) and integrated heats of injection (bottom panel) are presented for the titration between AcrIIA4 and *Spy*Cas9. In the bottom panel, the experimental data are shown as solid squares and the least squares best-fit curves derived from a simple one-site binding model are shown as a black line. The thermodynamic parameters for the complex formation are listed in Table 2.

We quantitatively measured the binding thermodynamics between AcrIIA4 and apo-*Spy*Cas9 using isothermal titration calorimetry (ITC). Our measurement revealed a large exothermic reaction between AcrIIA4 and *Spy*Cas9 with *K*_D_ = 4.8 ± 1.5 μM and a 1:1 stoichiometry (Fig. 4A). The interaction between AcrIIA4 and sgRNA bound-*Spy*Cas9 showed a significantly higher affinity, with the *K*_D_ value of 0.6 ± 0.1 nM (Fig. 4B). The binding affinity measured by ITC was similar to the previous report of *K*_D_ = 4.1 ± 1.1 nM from the microscale thermophoresis data^10^. Thus, the presence of sgRNA promoted the interaction between AcrIIA4 and *Spy*Cas9 by a factor of 8000 (Table 2). The weaker binding between AcrIIA4 and apo-*Spy*Cas9 explains previous observations that AcrIIA4 did not co-elute with *Spy*Cas9 in the absence of sgRNA from the size exclusion chromatography, though MBP pull-down assays showed a possible weak interaction between AcrIIA4 and apo-*Spy*Cas9^10–12^. We also note that AcrIIA4 did not exhibit measurable affinity to sgRNA alone (Figure S3). The binding of AcrIIA4 and *Spy*Cas9 was largely an enthalpy-driven reaction, which can be explained by their binding surfaces comprising extensive electrostatic interactions and hydrogen bonding networks^11^. The entropic contribution to the binding was unfavorable, which reflects a decrease in conformational flexibility at the interfaces upon complex formation. Indeed, the increased affinity of AcrIIA4 to sgRNA-bound *Spy*Cas9 largely originated from favorable entropic contribution, demonstrating that sgRNA binding to *Spy*Cas9 relieved the entropic cost of *Spy*Cas9 to accommodate AcrIIA4.

**Table 2:** Thermodynamic parameters for the interaction between AcrIIA4 and *Spy*Cas9 obtained by isothermal titration calorimetry at 25 °C.

Description	*K*_D_ (nM)	Δ*G* (kcal/mol)	Δ*H* (kcal/mol)	−*T*Δ*S* (kcal/mol)
AcrIIA4:*Spy*Cas9	4800 ± 150	−7.3 ± 0.1	−11.5 ± 0.7	4.2 ± 0.8
AcrIIA4:*Spy*Cas9‒sgRNA	0.6 ± 0.1	−12.6 ± 0.4	−10.3 ± 0.1	−2.3 ± 0.4

**Figure 5:**
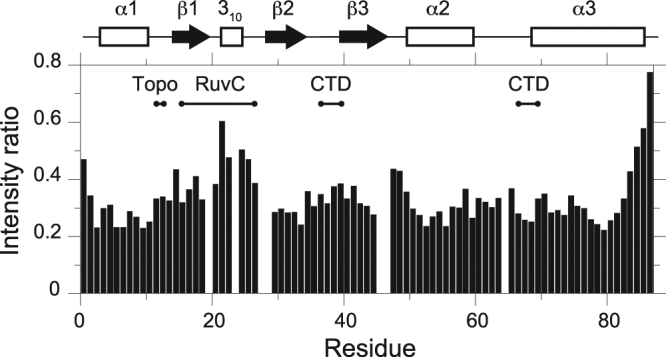
Intensity ratio of backbone amide resonances between AcrIIA4 in complex with *Spy*Cas9 (10% bound) and free AcrIIA4. The intensity ratio of backbone amide groups of AcrIIA4 measured from ^1^H–^15^N HSQC spectra at 25 °C is shown as a function of residue number. The intensity of 0.2 mM AcrIIA4 in free state (I_free_), and in complex with 0.02 mM *Spy*Cas9 (I_bound_) are measured and the ratio is presented as I_bound_/I_free_. The secondary structures of AcrIIA4 are shown on top as a schematic representation, and the binding interfaces of AcrIIA4 in the AcrIIA4‒*Spy*Cas9‒sgRNA complex (PDB code 5VW1) are shown above the ratio as a visual guidance.

^1^H‒^15^N HSQC spectra of ^15^N-AcrIIA4 titrated with unlabeled *Spy*Cas9 exhibited gradual line broadening without chemical shift changes. Due to the large molecular weight of *Spy*Cas9 (m.w. ~158.4 kDa), backbone amide resonances of free AcrIIA4 gradually disappeared without any sign of new signals from the complex, such that 0.2 mM ^15^N-AcrIIA4 complexed with *Spy*Cas9 at a 1:1 ratio resulted in complete line broadening, except for the C-terminal Asn87. Most amide resonances of free AcrIIA4 exhibited intensity reduction of >60%, when the complex formation reached 10% from the stoichiometric titration (Fig. 5). This indicates that the line broadening is caused not only by the large complex formation, but also by intermediate exchange from the interaction. It has been reported that the interaction surfaces in slow exchanging large macromolecular complexes can be identified by differential line broadening upon binding^19^. We examined the line broadening of individual residues of AcrIIA4 upon apo-*Spy*Cas9 binding. The line broadening was less severe at the terminal and internal loop regions, and interfacial residues such as Tyr67 and Asp69 exhibited larger line broadening than others. The differential line broadening, however, did not unambiguously identify the binding interface of AcrIIA4. Comparison of amide resonance intensities of AcrIIA4 between free and 10% bound states indicated that the intensity reduction spanned most backbone amide groups. We examined the line broadening upon titration at 10 °C and 45 °C, but could not distinguish the binding interfaces based on the differential line broadening. We infer that the dynamics of AcrIIA4 and *Spy*Cas9 in complex may have contributed to the intermediate exchange, obscuring the precise mapping of the binding interface.

The crystal structure of the AcrIIA4–*Spy*Cas9–sgRNA complex has shown that *Spy*Cas9 employs the RuvC domain, the TOPO domain, and the CTD to interact with AcrIIA4^10–12^. The interfaces at the TOPO domain and the CTD (*red spheres* in Figure S4) overlap with the PAM interaction site of *Spy*Cas9 for the target strand of the DNA substrate. The interface at the RuvC domain (*orange spheres* in Figure S4) is the active site pocket that cleaves the non-target strand of the double-stranded DNA substrate. When the binding interfaces in the complex are mapped onto apo‒*Spy*Cas9, interfaces from the RuvC domain and the CTD are relatively well maintained in apo‒*Spy*Cas9 (dashed circle in *red*; Figure S4), but those from the TOPO domain are completely lost (dashed circle in *black*; Figure S4). Based on the structural alignment, we speculate that AcrIIA4 binds to apo-*Spy*Cas9 in a similar manner to sgRNA-bound *Spy*Cas9, and that the RuvC domain and the CTD provide the interaction surfaces for AcrIIA4 in the absence of sgRNA. Binding of *Spy*Cas9 to sgRNA induces a major rearrangement of the helical REC domain, as well as ordering of the PAM interaction site at the TOPO domain and the CTD^20,21^. It is remarkable that the affinity of the AcrIIA4‒*Spy*Cas9 interaction increases by a thousand-fold upon sgRNA binding^10^. Thus, the guide RNA binding and concomitant conformational rearrangement of *Spy*Cas9 provide key interfaces not only for the DNA substrate but also for the inhibitor AcrIIA4.

It has been shown that AcrIIA4 competes with the double-strand DNA substrate for *Spy*Cas9 binding with a ~17-fold higher affinity^10^. AcrIIA4 inhibited the substrate DNA binding to *Spy*Cas9 in a concentration-dependent manner, but did not affect the dissociation of the DNA substrate from *Spy*Cas9, indicating a slow dissociation kinetics^12^. In other words, AcrIIA4 effectively inhibits the nuclease activity of *Spy*Cas9, but there is competition between the DNA substrate and AcrIIA4 targeting for the same interface on *Spy*Cas9. We demonstrated that AcrIIA4 bound sequentially to *Spy*Cas9 and then to guide RNA with increasing affinity. In the sequential binding mechanism, AcrIIA4 does not need to compete with target DNA for *Spy*Cas9 binding, since target DNA recognition requires a pre-formed assembly of guide RNA and *Spy*Cas9. The binary complex between AcrIIA4 and apo‒*Spy*Cas9 has a moderate affinity, but it can rapidly turn into a tight ternary complex in the presence of mature guide RNA, effectively preventing the target DNA binding. The sequential binding mechanism would be advantageous for the anti-CRISPR function, especially when the anti-CRISPR genes are integrated as a prophage, and constitutively inactivates the host CRISPR-Cas system^22^.

In summary, we have determined the solution structure of the anti-CRISPR protein AcrIIA4, and investigated the backbone dynamics using NMR spectroscopy. AcrIIA4 adopts a small and compact backbone fold, but exhibits significant dynamics in the loop region that serves as the binding interface for *Spy*Cas9. Notably, AcrIIA4 can bind to *Spy*Cas9 in the absence of sgRNA, and the binary complex can associate with sgRNA to form a tight ternary complex, avoiding competition with the DNA substrate for *Spy*Cas9 binding.

## Materials and Methods

### Cloning, protein expression, and purification

The synthetic AcrIIA4 (a.a. 1–87) gene was cloned into a modified pBT7-N-His vector (ATCC). The plasmid was introduced into *Escherichia coli* strain BL21star(DE3) cells (Invitrogen), and grown in LB or minimal media with ^15^NH_4_Cl and/or ^13^C_6_-glucose as sole nitrogen or carbon sources, respectively. Cells were grown at 37 °C to OD_600_ ~0.8, induced with 1 mM isopropyl-β-D-thiogalactopyranoside (IPTG) at 16 °C, and harvested by centrifugation after 16 hours of induction. Cell pellets were resuspended in 20 mM Tris-HCl, pH 7.4, 200 mM NaCl, 20 mM imidazole, and 1 mM phenylmethylsulfonylfluoride, lysed using Emulsiflex C3 (AVESTIN) and centrifuged at 20,000 g for 30 min. The clear supernatant was loaded onto a HisTrap column (GE Healthcare) equilibrated with 20 mM Tris-HCl, pH 7.4, 200 mM NaCl, and 20 mM imidazole, and eluted with 500 mM imidazole. The His_6_-tag was removed using TEV protease in 20 mM Tris-HCl, pH 8.0, 5 mM β-mercaptoethanol (BME), and 200 mM NaCl, and the digestion reaction was loaded onto the HisTrap column. The flow-through was collected and loaded onto a Superdex 75 column (GE Healthcare) equilibrated with 20 mM Tris-HCl, pH 7.4, 200 mM NaCl. Lastly, AcrIIA4 was loaded onto a MonoQ column (GE Healthcare) and eluted with a gradient of 0.1‒1 M NaCl.

The synthetic *S. pyogenes* Cas9 (a.a. 1–1368) gene was cloned into a pET28b vector with a C-terminal His_6_-tag, and transformed into *E. coli* strain BL21(DE3) cells. The cells were cultured in LB medium at 37 °C to OD_600_ ~0.7. Protein expression was induced by the addition of 0.5 mM IPTG, followed by incubation at 17 °C for 16 hours. Cells were harvested by centrifugation and resuspended in 20 mM Tris, pH 7.5, 300 mM NaCl, 5 mM BME, and 10% (w/v) glycerol. After sonication and centrifugation, the supernatant was loaded onto a 5 mL HisTrap HP column (GE Healthcare) pre-equilibrated with 20 mM Tris, pH 7.5, 300 mM NaCl, 5 mM BME, 10% (w/v) glycerol, and 30 mM imidazole. *Spy*Cas9 was eluted with a linear gradient of 450 mM imidazole and then dialyzed against 20 mM Tris-HCl, pH 7.5, 175 mM NaCl, 10% (w/v) glycerol, and 5 mM BME. The *Spy*Cas9 fraction was loaded onto a 5 mL HiTrap SP column (GE Healthcare) pre-equilibrated with the dialysis buffer. *Spy*Cas9 was eluted with a linear gradient of 1 M NaCl. The protein was further purified using a HiLoad 16/60 Superdex200 column (GE Healthcare) equilibrated with 20 mM Tris-HCl pH 8.0, 200 mM NaCl, 5% (w/v) glycerol, and 2 mM dithiothreitol.

### Preparation of sgRNA

The DNA template for RNA transcription was prepared by using the Giga-prep kit (Epigentics). The sgRNA was prepared *in vitro* by mixing rNTPs (rATP, rGTP, rCTP, and cUTP), MgCl_2_, T7 RNA polymerase (P266L mutant)^23^, inorganic pyrophosphatase, and the DNA template in the transcription buffer. After 6 hour transcription at 37 °C, the synthesized RNA was precipitated by ethanol treatment overnight, purified using 12% denaturing PAGE (19:1 cross-linking ratio), and electro-eluted (Elutrap, Whatman). The purified RNA was desalted and exchanged into water using Amicon (Millipore).

### NMR Spectroscopy

The NMR sample contained 2 mM ^13^C,^15^N-AcrIIA4 in 20 mM sodium phosphate, pH 7.4, 100 mM NaCl, 0.01% NaN_3_. NMR spectra were recorded at 25 °C on Bruker 600 MHz and 800 MHz spectrometers equipped with a *z*-shielded gradient triple resonance probe. Sequential and side chain assignments of ^1^H, ^15^N and ^13^C resonances were achieved by three-dimensional triple resonance through-bond scalar correlation experiments CBCA(CO)NH, HNCACB, HNCO, HN(CA)CO, HBHA(CO)NH, HCCH-TOCSY, and ^15^N-TOCSY-HSQC. 3D ^13^C-separated NOESY and ^15^N-separated NOESY experiments were obtained using a mixing time of 120 ms. The acquisition parameters for the 3D experiments are described in detail as Supplementary Information. The χ1 angle restraints were obtained based on the quantification of intraresidue NOEs between backbone amide proton, α proton, and β methylene protons^24^. Residual ^1^D_NH_ dipolar couplings were obtained by taking the difference in the J splitting values measured in oriented (10 mg/ml of *pf1* phage alignment media) and isotropic (water) AcrIIA4 using 2D in–phase/antiphase ^1^H‒^15^N HSQC spectra^25^. ^15^N-R_1_ and ^15^N-R_1_ρ relaxation rates, and heteronuclear ^1^H‒^15^N NOE were measured using pulse schemes described previously^26,27^ on the Bruker 800 MHz spectrometer. Delays of 10, 50, 100, 400, 800, 1200, 1500 ms were used for the R_1_ relaxation measurement, and 0, 4, 12, 24, 40, 60, 80, 96 ms used for R_1_ρ experiment. ^15^N-R_2_ relaxation rates were obtained from the ^15^N-R_1_ and ^15^N-R_1_ρ relaxation rates using the equation, R_2_ = (R_1_ρ − R_1_ sin^2^ θ)/cos^2^ θ, where θ = arctan(Ω_N_/γ_N_B_1_), Ω_N_ is the resonance offset, and γ_N_B_1_ is the spin-lock field strength^28^. For the heteronuclear ^1^H‒^15^N NOE measurement, ^1^H saturation was achieved using 3 s of 120° ^1^H pulses separated by 5 ms intervals. 2 s of relaxation delays were used for ^15^N-R_1_ and ^15^N-R_1_ρ relaxation measurements, and 4 s of the relaxation delay was used for the heteronuclear ^1^H‒^15^N NOE measurement. NMR spectra were processed using the NMRPipe^29^, and analyzed using PIPP^30^, and NMRView^31^ programs.

### Structure calculation

Interproton distance restraints were derived from the NOE spectra and classified into distance ranges according to the peak intensity. ϕ/ψ torsion angle restraints were derived from backbone chemical shifts using the program TALOS+^32^. The solution structure of AcrIIA4 was calculated by simulated annealing in torsion angle space using the Xplor-NIH program^13^. The target function of simulated annealing included a covalent geometry, a quadratic van der Waals repulsion potential^33^, square-well potentials for interproton distance and torsion angle restraints, hydrogen bonding, RDC restraints^34^, harmonic potentials for ^13^Cα/^13^Cβ chemical shift restraints^35^, a multidimensional torsion angle database potential of mean force^36^, and a radius of gyration term^37^. The radius of gyration represents a weak overall packing potential, and residues 1‒86 of AcrIIA4 were selected for the term. Structures were displayed using VMD-XPLOR software^38^. The violations from the structure calculation are described in detail as Supplementary Information.

### Isothermal Titration Calorimetry

The ITC experiment was performed at 25 °C using an iTC200 calorimeter (Malvern). To characterize the affinity of AcrIIA4 to *Spy*Cas9, 50 µM *Spy*Cas9 in the cell was titrated with 500 µM of the AcrIIA4 in 20 mM sodium phosphate, pH 7.4, 100 mM NaCl, 5 mM BME, and 0.01% NaN_3_. Twenty consecutive 2 μL aliquots of AcrIIA4 were titrated into the cell and the duration of each injection was 4 sec, with injections occurring at intervals of 150 sec. The heats associated with dilution of the substrates were subtracted from the measured heats of binding. ITC titration data were analyzed using the Origin version 7.0 program provided with the instrument.

### Data availability

Atomic coordinates and NMR restraints of the reported solution structure have been deposited in the Protein Data Bank under accession code 5XN4 and in the Biological Magnetic Resonance Bank under accession code 36085, respectively.

## Electronic supplementary material

Supplementary Information
