# Supplementary material for: Solution structure and dynamics of anti-CRISPR AcrIIA4, the Cas9 inhibitor
Source: Sci Rep. 2018 Mar 1;8:3883. doi: 10.1038/s41598-018-22177-0 (PMC5832863; doi:10.1038/s41598-018-22177-0)

Supplementary Information

Solution structure and dynamics of anti-CRISPR AcrIIA4, the Cas9 inhibitor

Iktae Kim[a], Migyeong Jeong[a], Donghyun Ka[a], Mookyoung Han[a], Nak-Kyoon Kim[b], Euiyoung Bae[a], and Jeong-Yong Suh[a,c,1]

[a]Department of Agricultural Biotechnology and Research Institute of Agriculture and Life Sciences, Seoul National University, Seoul 08826; [b]Advanced Analysis Center, Korea Institute of Science and Technology, Seoul 02792, Korea; [c]Institute for Biomedical Sciences, Shinshu University, Minamiminowa, Nagano 399-4598, Japan

1. Experimental parameters for 3D experiments

3D experiments	^{1}H sw	^{15}N sw	^{15}N o3p	^{15}N td	^{13}C sw	^{13}C o2p	^{13}C td	^{1}H sw	^{1}H td	ns
CBCA(CO)NH	12	31	120.5	64	75	43	128			4
HNCACB	12	31	120.5	64	75	43	128			8
HNCO	12	31	120.5	64	12	176	64			8
HN(CA)CO	12	31	120.5	64	12	176	64			8
HBHA(CO)NH	12	31	120.5	64				5	128	4
HCH-TOCSY	12				75	43	64	8	128	4
CCH-TOCSY	12				75	43	64			4
15N-TOCSY-HSQC	12	31	120.5	64				12	128	4
15N-separated NOESY	12	31	120.5	64				12	256	4
13C-separated NOESY	12				75	43	88	12	128	4

sw: spectral width; o2p and o3p: carrier frequencies for ^{13}C and ^{15}N nuclei; td: time domain data points; ns: number of scans

2. Violations in the structure calculation

1) Violations statistics for NOE (threshold: 0.5) in 20 structures:

atom-1		atom-2		viols	ave.delta	max.delta
45	HN	50	HN	6	0.5436	0.5652

2) Violations statistics for CDIH (threshold: 5) in 20 structures:

atom-1		atom-2		atom-3		atom-4		viols	ave.delta	max.delta
66	N	66	CA	66	C	67	N	6	5.9432	6.8010
76	N	76	CA	76	C	77	N	3	-6.7673	-6.9100
21	C	22	N	22	CA	22	C	1	-5.6700	-5.6700
49	C	50	N	50	CA	50	C	1	5.0020	5.0020
75	C	76	N	76	CA	76	C	1	5.1910	5.1910

Supplementary Figure 1. Size exclusion chromatograms using a Superdex 75 26/600 PG column (GE Healthcare, USA) for AcrIIA4. Elution profiles of standard marker proteins are shown at the top of the chromatogram as a reference.

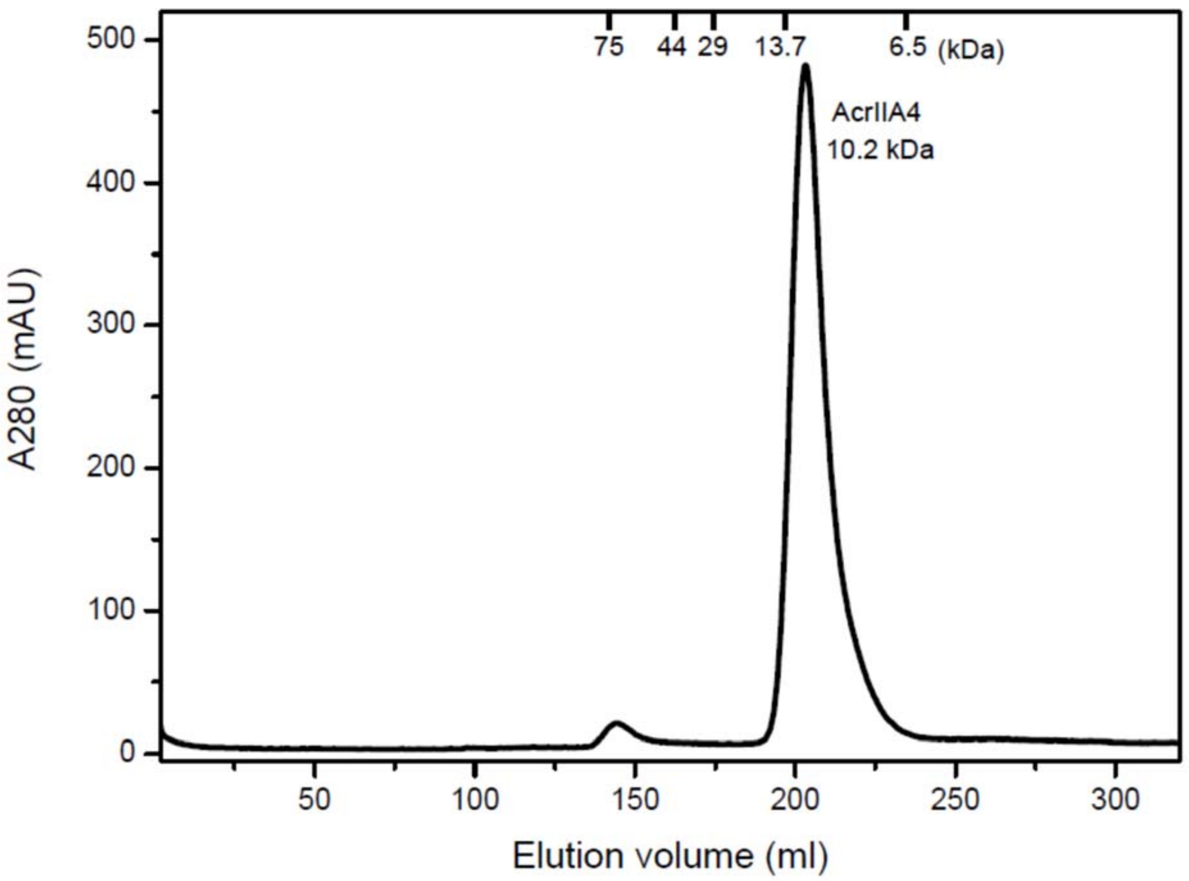

Supplementary Figure 2. ^{1}H–^{15}N HSQC spectra of 2 mM AcrIIA4 in 20 mM sodium phosphate, pH 7.4, 100 mM NaCl, and 0.01% NaN_3. The backbone amide resonances are annotated with the residue types and numbers.

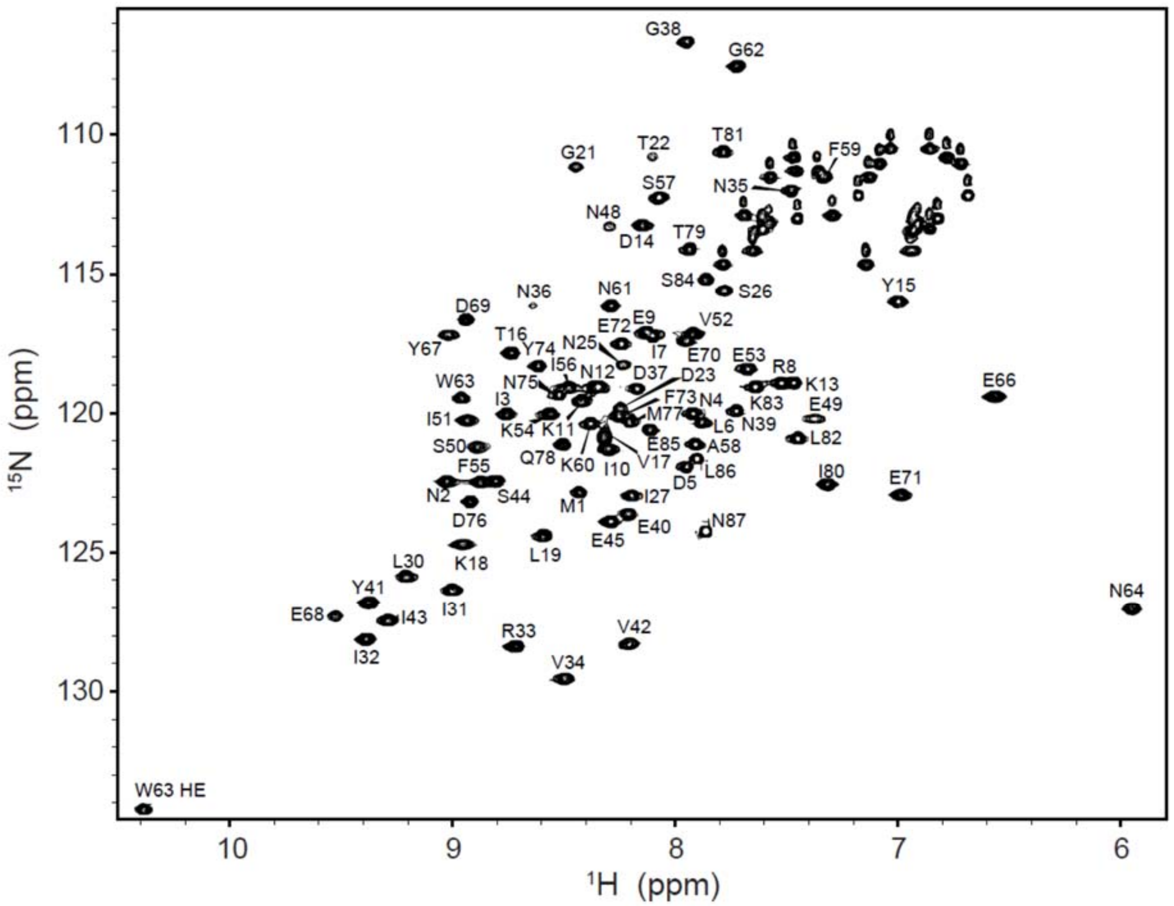

Supplementary Figure 3. ITC for the interaction between AcrIIA4 and sgRNA. Raw ITC data (Top panel) and integrated heats of injection (bottom panel) are presented for the titration between AcrIIA4 and sgRNA. The lack of characteristic binding isotherm indicates that AcrIIA4 did not bind to sgRNA alone.

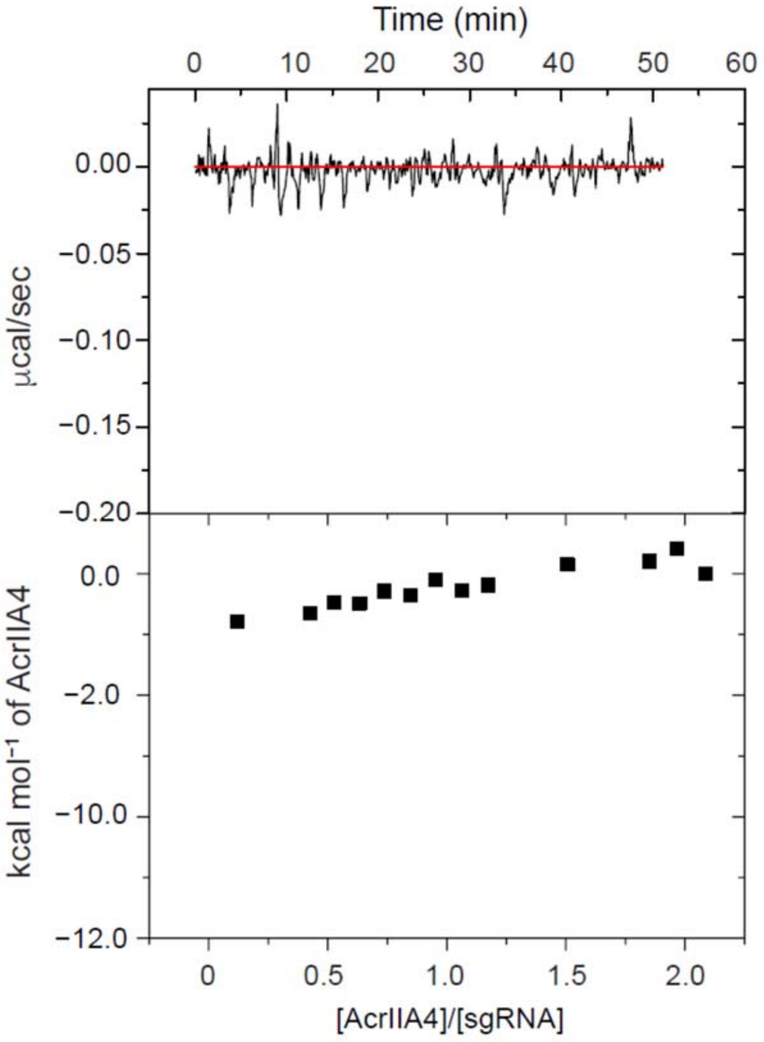

Supplementary Figure 4. *Top* panel: Domain organization of *Spy*Cas9 comprising the RuvC nuclease domain, the bridge helix (BH), the RNA recognition domain (REC), the HNH nuclease domain, the topoisomerase-homology domain (TOPO), and the C-terminal domain (CTD). *Bottom left* panel: The crystal structure of the AcrIIA4–*Spy*Cas9–sgRNA complex (PDB code 5VW1) in a ribbon diagram. Individual domains of *Spy*Cas9 are colored according to the color scheme of the domain organization. AcrIIA4 is colored in *light blue*, and sgRNA is colored in *magenta*. The interfacial residues of *Spy*Cas9 for AcrIIA4 are shown in a space-filling model, and colored in *red* (PAM recognition site) or in *orange* (RuvC nuclease site). Interfaces from the CTD and the RuvC domain are highlighted by dashed circles in *red*, and interfaces from the TOPO domain are highlighted by dashed circles in *black*. *Bottom right* panel: The interfacial residues of *Spy*Cas9 for AcrIIA4 are mapped on to the crystal structure of apo-*Spy*Cas9 (PDB code 4CMP) in a space-filling model. The domains are colored according to the same color scheme as in the complex structure.

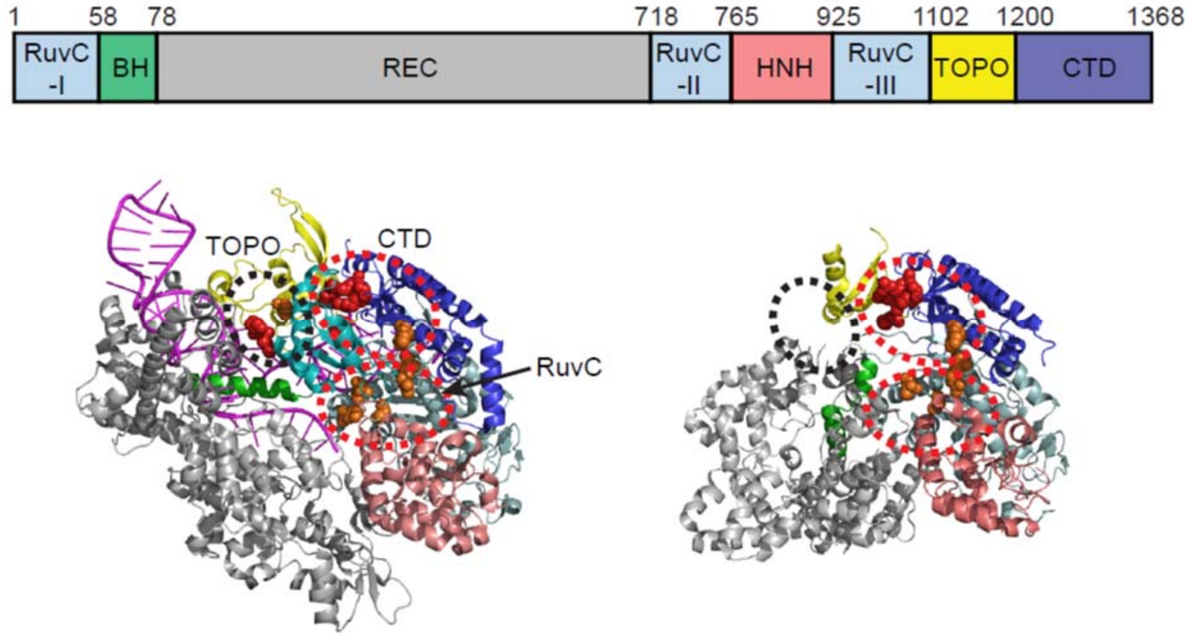

Supplement: Supplementary file 1 — Supplementary Information [file 41598_2018_22177_MOESM1_ESM.pdf]
